# Reduced Sensitivity of Commercial Spike-Specific Antibody Assays after Primary Infection with the SARS-CoV-2 Omicron Variant

**DOI:** 10.1128/spectrum.02129-22

**Published:** 2022-08-25

**Authors:** David Niklas Springer, Thomas Perkmann, Claudia Maria Jani, Patrick Mucher, Katja Prüger, Rodrig Marculescu, Elisabeth Reuberger, Jeremy Vann Camp, Marianne Graninger, Christian Borsodi, Josef Deutsch, Oliver Lammel, Stephan Walter Aberle, Elisabeth Puchhammer-Stöckl, Helmuth Haslacher, Eva Höltl, Judith Helene Aberle, Karin Stiasny, Lukas Weseslindtner

**Affiliations:** a Center for Virology, Medical University of Vienna, Vienna, Austria; b Department of Laboratory Medicine, Medical University of Vienna, Vienna, Austria; c Private Practice, Völkermarkt, Austria; d Private Practice, Ramsau am Dachstein, Austria; e Center for Public Health, Medical University of Vienna, Vienna, Austria; University of Siena

**Keywords:** SARS-CoV-2, Omicron, antibodies, neutralization, antibody assay, sensitivity, surrogate assay, immunoassay

## Abstract

The SARS-CoV-2 Omicron variant is characterized by substantial changes in the antigenic structure of the Spike (S) protein. Therefore, antibodies induced by primary Omicron infection lack neutralizing activity against earlier variants. In this study, we analyzed whether these antigenic changes impact the sensitivity of commercial anti-SARS-CoV-2 antibody assays. Sera from 37 unvaccinated, convalescent individuals after putative primary Omicron infection were tested with a panel of 20 commercial anti-SARS-CoV-2 immunoassays. As controls, we used samples from 43 individuals after primary infection with the SARS-CoV-2 ancestral wild-type strain. In addition, variant-specific live-virus neutralization assays were used as a reference for the presence of SARS-CoV-2-specific antibodies in the samples. Notably, in Omicron convalescents, there was a statistically significant reduction in the sensitivity of all antibody assays containing S or its receptor-binding-domain (RBD) as antigens. Furthermore, antibody levels quantified by these assays displayed a weaker correlation with Omicron-specific neutralizing antibody titers than with those against the wild type. In contrast, the sensitivity of nucleocapsid-protein-specific immunoassays was similar in wild-type and Omicron-infected subjects. In summary, the antigenic changes in the Omicron S lead to reduced immunoreactivity in the current commercial S- and RBD-specific antibody assays, impairing their diagnostic performance.

**IMPORTANCE** This study demonstrates that the antigenic changes of the SARS-CoV-2 Omicron variant affect test results from commercial Spike- and RBD-specific antibody assays, significantly diminishing their sensitivities and diagnostic abilities to assess neutralizing antibodies.

## INTRODUCTION

The SARS-CoV-2 Omicron variant, which emerged in late 2021 (1), displays more than 30 mutations in the gene coding for the Spike (S) protein, leading to substantial changes in the antigenic structure in particular in the receptor-binding domain (RBD), the main target for neutralizing antibodies (nAbs) (2–4).

As an effect of these alterations, there was a significant decrease in the neutralizing capability of preexisting antibodies induced by prior infection with other variants or vaccinations (3, 5–7). In contrast, antibodies produced after primary infection with the Omicron variant were recently found to have limited neutralizing activity against earlier variants, including the wild-type and Delta variant (8, 9).

While neutralization tests (NTs) could be rapidly adapted by using clinical isolates or pseudoviruses (8, 9), most commercial antibody assays have not been modified so far (10). However, such adaptations could be required because most of these assays had been developed before the emergence of variants of concern (VOCs) and contain the S or RBD protein as target antigens derived from the ancestral wild type (WT) isolated in Wuhan (10).

Commonly used antibody tests include enzyme-linked immunosorbent assays (ELISA), chemiluminescence immunoassays (CLIAs), and immunoblots (IBLs) (11–13), often standardized by the World Health Organization (WHO) measuring binding antibody units per milliliter (BAU/mL) (14). In addition, surrogate virus neutralization tests (sVNTs) are in use, quantifying the antibody-mediated inhibition of binding of the RBD to the angiotensin-converting enzyme 2 (ACE2) as a correlate for neutralization (12, 15, 16).

Therefore, the question has arisen whether Omicron S- and Omicron RBD-specific antibodies bind less efficiently to the antigens used in these commercial antibody assays (10). In the present study, we analyzed serum samples from 37 nonhospitalized individuals with putative Omicron primary infection in a panel of 20 commercial SARS-CoV-2 antibody assays. The detection rates of the assays were compared with those obtained with a matched control cohort of 43 nonhospitalized convalescents after WT primary infection. In addition, since the nucleocapsid protein’s (NC) structure is mainly preserved in the Omicron variant (2), we included assays containing NC as antigen as a further control.

## RESULTS

### Characteristics and matching of SARS-CoV-2 convalescent individuals.

**FIG 1:**
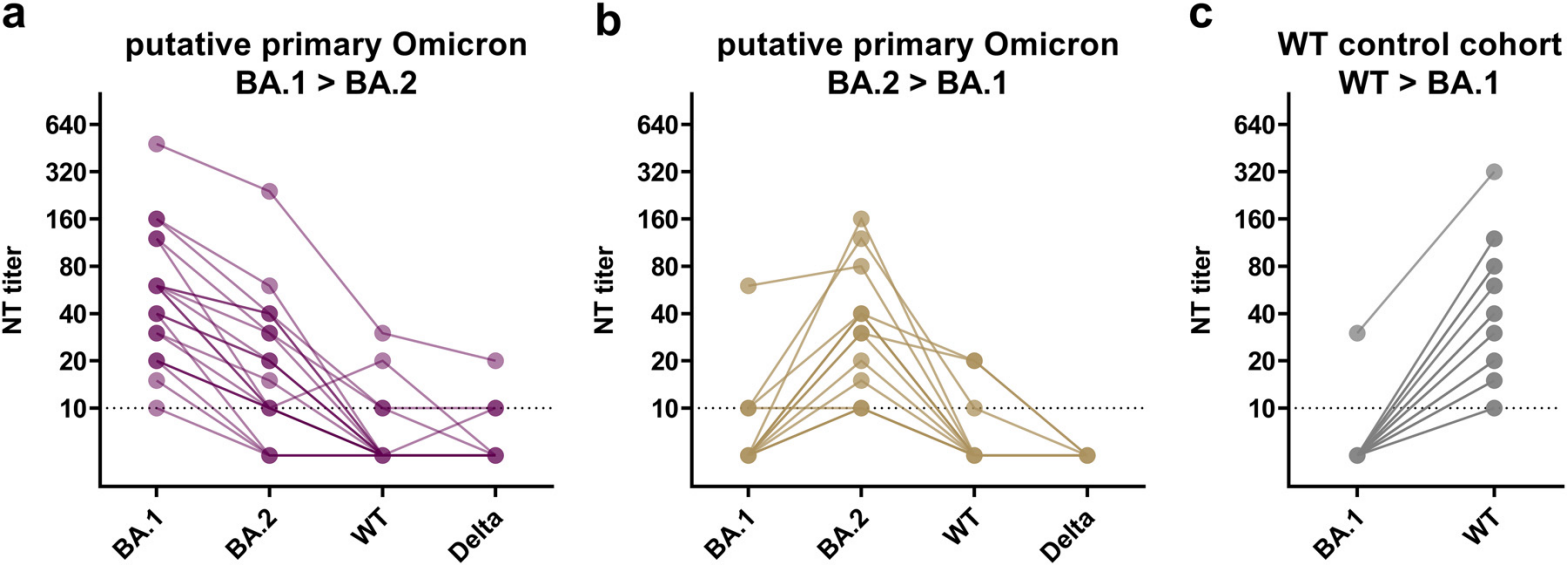
Cross-neutralizing activity in serum samples from convalescents after putative primary Omicron infection and infection with WT virus (control cohort) against BA.1, BA.2, Delta, and an ancestral WT strain. All samples from convalescents after putative primary infection with the Omicron variant (*n* = 37) display higher neutralizing activity against one of the tested Omicron sublineages (BA.1, BA.2) than against earlier variants (Delta, WT). Titers from individual samples are connected with lines. Samples with a higher neutralizing titer against BA.1 than against BA.2 (*n* = 22) are displayed in violet (a). Samples with higher titers against BA.2 than against BA.1 (*n* = 15) are displayed in brown (b). Inversely to convalescents after putative primary Omicron infection, neutralizing titers against the ancestral WT strain were significantly higher in the convalescents after infection with the WT virus (control cohort, *n* = 43). Neutralizing titers from convalescents after WT infection are shown in gray (c). The figure illustrates data from 29 convalescents after putative primary Omicron infection described in detail by Medits et al. (9).

The study included serum samples from 37 nonhospitalized, unvaccinated convalescents after primary infection with the Omicron variant. In all 37 individuals, a positive RT-PCR result from a nasopharyngeal swab preceded the acquisition of the respective serum sample (median interval between RT-PCR positivity and acquisition of the serum sample: 33 days, range:16 to 96). The swabs were obtained during a period when the Omicron sublineages BA.1 or BA.2 circulated in Austria with over 98% predominance (17). Furthermore, the samples of these individuals displayed significantly higher BA.1- or BA.2-specific titers of neutralizing antibodies (nAbs) than against a WT strain with the D614G mutation (B.1.1) and the Delta variant of concern (VOC) in live-virus NTs, as demonstrated previously (Fig. 1a and b) (9).

Serum samples from 43 nonhospitalized convalescents after infection with WT virus early in the pandemic (before the emergence of VOCs, February 2020 to December 2020) served as controls. RT-PCR-positivity in controls preceded the collection of the respective serum samples with a median interval of 35 days (range: 16 to 70). Inversely to convalescents after putative primary Omicron infection, titers of nAbs against the ancestral WT strain were significantly higher in these individuals than titers against the Omicron variant (Fig. 1c).

Samples from WT controls were matched to those from convalescents after Omicron infection based on the concentration of variant-specific nAbs, age, the interval between PCR diagnosis and serum sampling, and the absence of hospitalization. Fig. S1a shows that the matched groups of convalescents after Omicron and WT infections exhibited comparable virus-specific neutralization titers (BA.1/BA.2 versus WT titers: *P* = 0.42, two-tailed Mann-Whitney U test; Fig. S1a). In addition, there was no difference in age (*P* = 0.32; Fig. S1b) or the interval between PCR-positivity and the time point when serum samples were obtained (*P* = 0.86, two-tailed Mann-Whitney U test, respectively; Fig. S1c).

### Detection rates of commercial antibody assays in convalescents after Omicron infection.

Serum samples from matched groups of convalescents after putative primary Omicron (*n* = 37) and WT (*n* = 43) infection were tested using a panel of 20 commercial antibody assays by seven manufacturers. Detailed information on the antibody assays, including test principle, target antigens, measuring units, covered immunoglobulin class, and cutoff values, are provided in Table S1.

**FIG 2:**
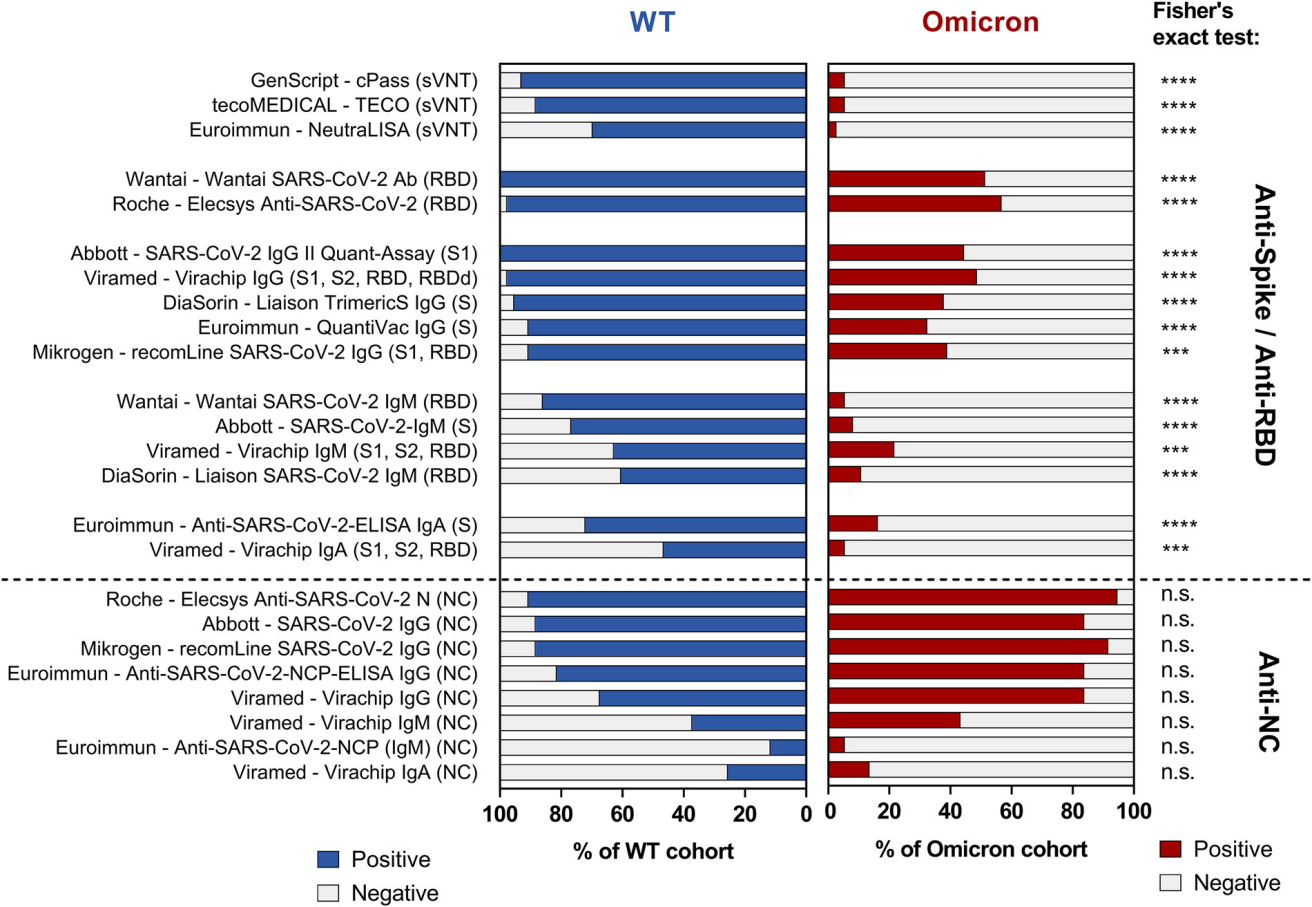
Detection rates of anti-spike (S)-, anti-Receptor-binding-domain (RBD)- and anti-nucleocapsid (NC) antibody assays in convalescents after primary infection with SARS-CoV-2 wild type (WT) and the Omicron variant. Graphical representation of the detection rates (percentage of the samples tested positive) in the anti-SARS-CoV-2 antibody assays. Blue: Control (WT) cohort (*n* = 43); red: Omicron cohort (*n* = 37), except for Mikrogen – recomLine (S1, RBD, NC) (one sample technically invalid) and Abbott – SARS-CoV-2 IgG II Quant Assay (not sufficient sample material), *n* = 36, respectively. Asterisks (*) indicate a significant difference in two-tailed Fisher's exact test after correction for multiple testing (alpha = 0.05). [*, *P* < 0.05; **, *P* < 0.01; ***, *P* < 0.001; ****, *P* < 0.0001; n.s.: not significant (*P* > 0.05)]. The immunoassays are denoted as “company – test kit name (target antigen)”; The immunoblots and microarrays (recomLine IgG, Virachip IgG/IgA/IgM) are listed twice as the S/RBD- and NC-signals were analyzed separately. Detailed information on the evaluated immunoassays is provided in Table S1 in the supplemental material.

As shown in Fig. 2, we observed significantly reduced detection rates in all commercial antibody assays based on S or RBD as target antigens with samples from convalescents after putative primary Omicron infection compared to the WT control group (*P* < 0.05, two-tailed Fisher’s exact test, Bonferroni correction for multiple testing).

In contrast, NC-based assays displayed no significant differences in the detection rates among convalescents after Omicron and WT infection (*P* > 0.05 in all assays). Consequently, the detection rates of the two IBLs (SARS-CoV-2 ViraChip IgG by Viramed and recomLine SARS-CoV-2 IgG by Mikrogen) were also similar among the groups (*P* > 0.05) since both IBLs combine the signal of S-, RBD- and NC-specific antibody testing into a single test result.

The detailed results for all assays, including absolute and relative detection rates and comparative analyses, are displayed in Table 1.

**TABLE 1:** Detection rates of commercial antibody assays in SARS-CoV-2 wild-type (WT) and Omicron primary infections^*a*^

Target antibodies	Assay		Principle	Target antigen(s)	WT		Omicron		Fisher
					n	%	n	%	*P* value
Anti-S-/Anti-RBD antibody assay	Surrogate Virus Neutralization Test (sVNT)								
	IgG/A/M	cPass SARS-CoV-2 Neutralization Antibody Detection kit (GenScript)	sVNT	RBD-ACE2 Inhibition	40/43	93%	2/37	5%	**<0.0001**
		TECO SARS-CoV-2 Neutralization Antibody Assay (TECOmedical)			38/43	88%	2/37	5%	**<0.0001**
		SARS-CoV-2-NeutraLISA (Euroimmun)			30/43	70%	1/37	3%	**<0.0001**
	Anti-S-Total Antibody Tests								
	IgG/A/M	WANTAI SARS-CoV-2 Ab Elisa (Wantai)	ELISA	RBD	43/43	100%	19/37	51%	**<0.0001**
		Elecsys Anti-SARS-CoV-2 S (Roche)	ECLIA		42/43	98%	21/37	57%	**0.0003**
	Anti-S-IgG/Anti-RBD-IgG								
	IgG	SARS-CoV-2 IgG II Quant-Assay (Abbott)	CMIA	S	43/43	100%	16/36	44%	**<0.0001**
		SARS-CoV-2 Virachip IgG (Viramed)	MA	S1+S2+RBD+RBDd	42/43	98%	18/37	49%	**<0.0001**
		LIAISON SARS-CoV-2 TrimericS IgG assay (DiaSorin)	CLIA	S	41/43	95%	14/37	38%	**<0.0001**
		Anti-SARS-CoV-2-QuantiVac-ELISA (Euroimmun)	ELISA		39/43	91%	12/37	32%	**<0.0001**
		recomLine SARS-CoV-2 IgG (Mikrogen)	IB	S1+RBD	39/43	91%	14/36	39%	**<0.0001**
	Anti-S-IgM/Anti-RBD-IgM								
	IgM	WANTAI SARS-CoV-2 IgM Elisa (Wantai)	ELISA	RBD	37/43	86%	2/37	5%	**<0.0001**
		SARS-CoV-2 IgM (Abbott)	CMIA	S	33/43	77%	3/37	8%	**<0.0001**
		SARS-CoV-2 Virachip IgM (Viramed)	MA	S1+S2+RBD	27/43	63%	8/37	22%	**0.0078**
		LIAISON SARS-CoV-2 IgM (DiaSorin)	CLIA	RBD	26/43	60%	4/37	11%	**<0.0001**
	Anti-S-IgA/Anti-RBD-IgA								
	IgA	Anti-SARS-CoV-2-ELISA (IgA) (Euroimmun)	ELISA	S	31/43	72%	6/37	16%	**<0.0001**
		SARS-CoV-2 Virachip IgA (Viramed)	MA	S1+S2+RBD	20/43	47%	2/37	5%	**0.0009**
Anti-NC-antibody assay	Anti-NC-Antibody Assays (IgG, IgA, IgM)								
	IgG/A/M	Elecsys Anti-SARS-CoV-2 N (Roche)	ECLIA	NC	39/43	91%	35/37	95%	0.6809
	IgG	SARS-CoV-2 IgG (Abbott)	CMIA		38/43	88%	31/37	84%	0.7464
		recomLine SARS-CoV-2 IgG (Mikrogen)	IB		38/43	88%	32/36	89%	>0.999
		Anti-SARS-CoV-2-NCP-ELISA (IgG) (Euroimmun)	ELISA		35/43	81%	31/37	84%	>0.999
		SARS-CoV-2 Virachip IgG (Viramed)	MA		29/43	67%	31/37	84%	0.1223
	IgM	SARS-CoV-2 Virachip IgM (Viramed)			16/43	37%	16/37	43%	0.6504
		Anti-SARS-CoV-2-NCP-ELISA (IgM) (Euroimmun)	ELISA		5/43	11%	2/37	5%	0.4416
	IgA	SARS-CoV-2 Virachip IgA (Viramed)	MA		11/43	26%	5/37	14%	0.2629
Mixed	Overall interpretation (Immunoblot, Microarray)								
	IgG	SARS-CoV-2 Virachip IgG (Viramed)	MA	S1+S2+RBD+RBDd+NC	42/43	98%	32/37	87%	0.0904
	IgM	SARS-CoV-2 Virachip IgM (Viramed)		S1+S2+RBD+NC	24/43	56%	6/37	16%	**0.0118**
	IgA	SARS-CoV-2 Virachip IgA (Viramed)			19/43	44%	2/37	5%	**0.0022**
	IgG	recomLine SARS-CoV-2 IgG (Mikrogen)	IB	S1+RBD+NC	41/43	95%	33/36	92%	>0.999

a N, number of positive samples/number of samples tested; %, percentage of positive samples; Fisher, Fisher’s exact test (two-tailed, alpha level = 0.05, all significant *P* values Bonferroni-adjusted for multiple testing assuming 28 tests); sVNT, SARS-CoV-2 Surrogate Virus Neutralization Tests; ELISA, enzyme-linked immunosorbent assay; CLIA, chemiluminescence immunoassay; CMIA, chemiluminescence micro particle assay; ECLIA, electrochemiluminescence immunoassay; IB, immunoblot; S, spike; S1/S2, subunit 1/2 of spike; RBD, receptor-binding-domain; RBDd, receptor binding domain of the Delta variant; NC, nucleocapsid; MA, microarray; WT, *n* = 43; Omicron, *n* = 37; except for recomLine SARS-CoV-2 IgG (Mikrogen) and SARS-CoV-2 IgG II Quant-Assay (Abbott), where Omicron *n* = 36 (one sample not technically valid and no sample material left, respectively). Significant *p*-values (*P* < 0.05) are bold, underlined. The immunoblots (Virachip IgG/IgA/IgM and the recomLine IgG) are listed three times as a separate analysis of the anti-S/RBD-, anti-NC-, and the overall-detection rate was calculated.

### Detection rates in subgroups of primarily Omicron-infected convalescents.

In nine out of the 37 individuals with putative primary Omicron infection (as epidemiologically indicated by the time of infection between January and March 2022), a variant-specific PCR was additionally performed. This PCR confirmed infection with the Omicron variant and identified the sublineages BA.1 in four and BA.2 in five individuals. Thus, we analyzed whether the detection rates of the commercial antibody assays were also reduced in this particular subgroup of convalescents. Analogously to the whole cohort of individuals with putative Omicron infection, the detection rates of S- and RBD-specific immunoassays were also reduced in these nine individuals with Omicron infection confirmed by a variant-specific PCR (reduced detection rates in 13 out of 16 evaluated assays). Again, the NC-specific antibody assays displayed similar detection rates among these nine individuals and convalescents after WT infection (Table S2).

To clarify whether the reduction in the detection rates of the assays was due to different antibody concentrations among the two groups, we calculated the diagnostic performances using only samples from Omicron and WT convalescents with NT titers ≥20 against the respective variant (Omicron: *n* = 30, WT *n* = 35). Indeed, the commercial antibody assays’ detection rates were also significantly reduced in samples with overall high antibody concentrations (all anti-S/anti-RBD assays *P* < 0.05; all anti-NC assays *P* > 0.05; Fisher’s exact test; Table S3).

### Correlation of quantitative antibody levels and variant-specific nAb titers.

**FIG 3:**
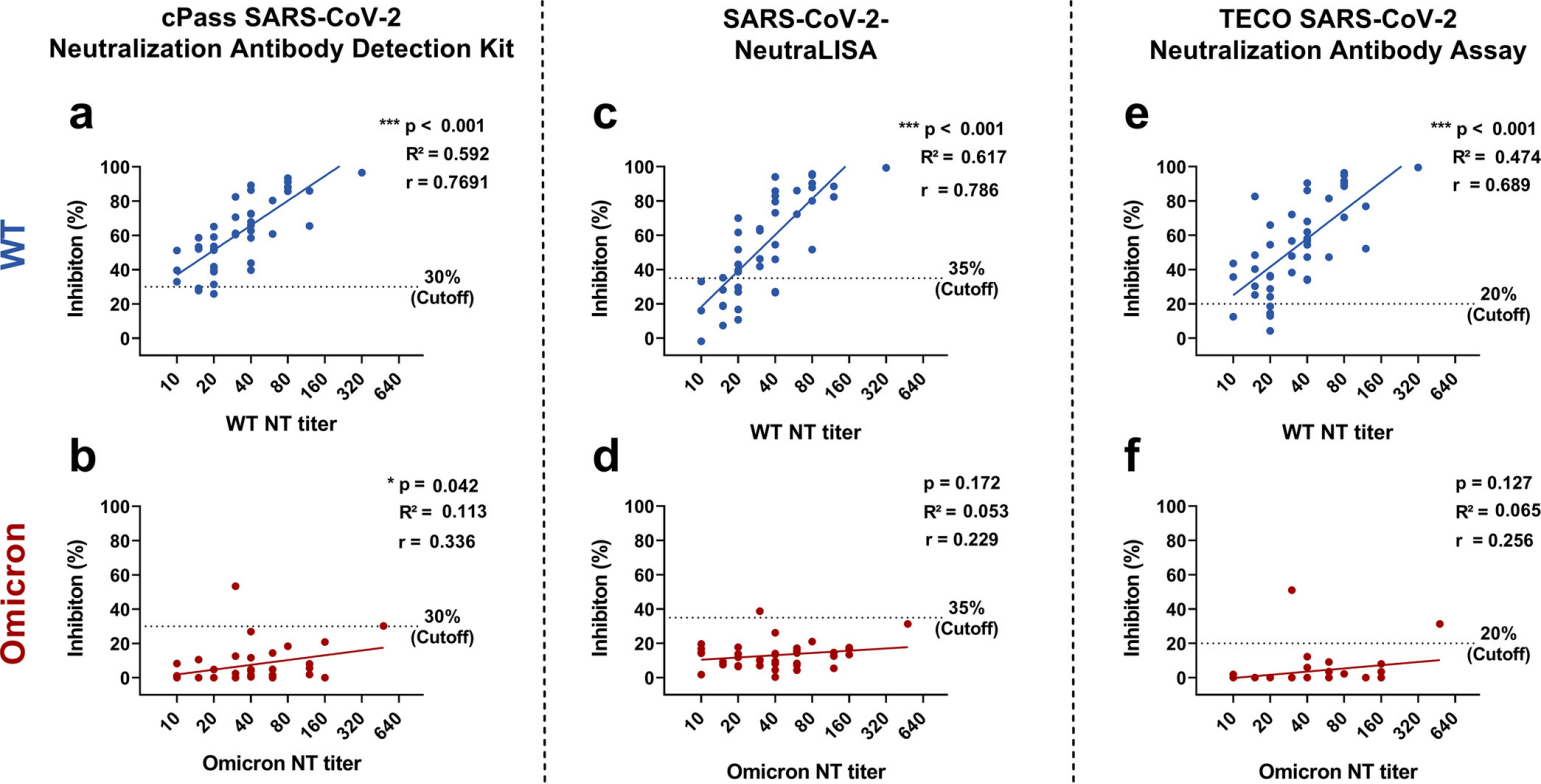
Correlation between antibody levels in SARS-CoV-2 Surrogate Virus Neutralization Tests (sVNTs) and variant-specific NT titers in SARS-CoV-2 wildtype (WT) and Omicron primary infections. Graphical description of the correlation and the linear regression of the results of the surrogate virus neutralization tests (as % RBD-ACE2-binding-inhibition) with respective quantitative titers of variant-specific NTs (Omicron, WT; both in log transformation). a) cPass versus WT NT, b) cPass versus Omicron NT, c) NeutraLISA versus WT NT, d) NeutraLISA versus Omicron NT, e) TECO versus WT NT, f) TECO versus Omicron NT. Dashed lines indicate the cutoff as recommended by the manufacturer. Blue dots: WT cohort (*n* = 43); red dots: Omicron cohort (*n* = 37). *P*-values, correlation coefficients r and R^2^ were calculated using Pearson correlation. Asterisks (*) indicate a significant correlation. (*, *P* < 0.05; **, *P* < 0.01; ***, *P* < 0.001).

Finally, we analyzed the correlation between the quantitive titers of nAbs against the variant the infection occurred with and the antibody levels quantified by the commercial antibody assays. Fig. 3 shows a robust correlation between RBD-ACE2 binding inhibition quantified by commercial sVNTs and the respective NT titers in convalescent-phase samples obtained from WT-infected subjects (*r* = 0.7 to 0.8). In contrast, a much weaker correlation was observed in sera from Omicron-infected subjects (*r* = 0.2 to 0.3), as indicated by a flattened steepness of the regression line (Fig. 3).

**FIG 4:**
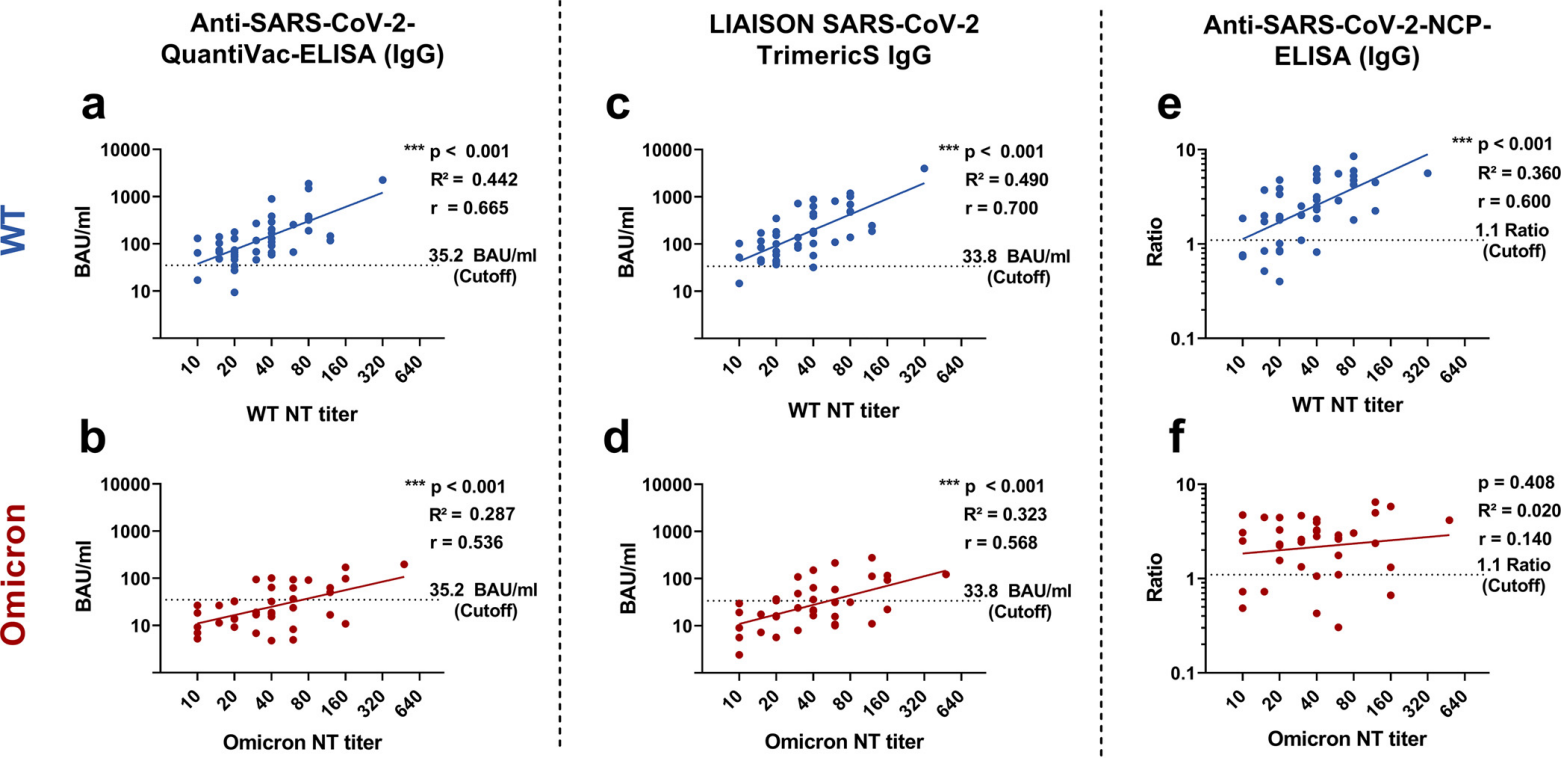
Correlation of anti-Spike (S)- and anti-nucleocapsid (NC) antibody levels with variant-specific NT titers in SARS-CoV-2 wild type (WT) and Omicron primary infections. Graphical description of the correlation and the linear regression of the quantitative results by selected ELISAs and a CLIA (BAU/mL) to the respective titers of variant-specific NTs (Omicron, WT; both in log transformation). a) QuantiVac by Euroimmun: versus WT NT b) QuantiVac by Euroimmun: versus Omicron NT c) Liaison TrimericS IgG by DiaSorin versus WT NT d) Liaison TrimericS IgG by DiaSorin versus Omicron e) Anti-SARS-CoV-2-NCP-ELISA (IgG) by Euroimmun versus WT NT) f) Anti-SARS-CoV-2-NCP-ELISA (IgG) by Euroimmun versus Omicron NT) a–d: anti-S IgG assays; e–f: anti-NC IgG antibody assay. Dashed lines indicate the cutoff as recommended by the manufacturer. Blue dots: WT cohort (*n* = 43); red dots: Omicron cohort (*n* = 37). *P*-values, correlation coefficients r and R^2^ were calculated using Pearson correlation (*, *P* < 0.05; **, *P* < 0.01; ***, *P* < 0.001). Data on the correlations of the other commercial antibody assays are provided in the Supplement (Supplementary Fig. S2–S9).

The S- and RBD-specific ELISAs, CLIAs, and the IBLs displayed an overall reduction in the signal intensity, i.e., the regression lines in the Omicron cohort shifted downwards (Fig. 4a to d, Supplementary Fig. S2 to S8). In contrast to Anti-S- and Anti-RBD-immunoassays, the signal intensities were comparable for NC-specific antibody assays among both cohorts (Fig. 4e and f, Supplementary Fig. S2e, S3c, S5d, S7d, and S9).

## DISCUSSION

The antigenic changes in the S protein of the SARS-CoV-2 Omicron variant cause a significant reduction in the neutralizing activity of preexisting antibodies induced by infections with earlier virus variants or vaccinations (5–7, 18, 19). Furthermore, nABs that are produced upon primary infection with the Omicron variant specifically neutralize the respective BA.1 or BA.2 subtypes but lack neutralizing activity against the WT and the Delta VOC (9). In this study, we analyzed whether the changes of the Omicron S protein affect the sensitivity of commercial antibody assays.

Indeed, we demonstrate significantly reduced detection rates in a large panel of commercial S- and RBD-specific antibody tests in convalescent individuals after putative primary Omicron infection. This finding, together with a decreased correlation between nAb titers and antibody levels by S- or RBD-specific commercial assays, indicates that antibodies against the S protein of the Omicron variant bind less efficiently to the S protein of the ancestral SARS-CoV-2 wild type used as target antigen in these assays. Furthermore, we found that detection rates in Omicron-convalescents did not decrease in antibody assays containing NC as antigen, which is mainly preserved in the Omicron variant (2).

Interestingly, the three sVNTs included in our panel of antibody assays were the most significantly affected assays by the mismatch between Omicron-specific antibodies and the original RBD as the target antigen. As shown in Fig. 3, the correlation between the sVNT results and the NT titer levels was noticeably weaker in the Omicron cohort than in the WT cohort. Of note, not even in samples with the highest Omicron-specific NT titers, sVNTs detected any significant RBD-ACE2-binding-inhibition. These findings can be explained by the accumulation of mutations in the RBD of the Omicron variants, with most mutations occurring at the RBD-ACE2 binding region (2–4). Antibodies formed against the Omicron RBD are thus less likely to inhibit the binding of the original RBD to ACE2 in the test. In the light of the emerging Omicron BA.4 and BA.5 sublineages with additional mutations in RBD of the Omicron BA.4 and BA.5 sublineages (20), further evaluations for such assay formats are required.

The current clinical use of SARS-CoV-2-specific antibody assays includes the identification of recent and past SARS-CoV-2 infections in patients presenting with potential post-COVID sequelae (e.g., Multi Inflammatory Syndrome in children (21, 22), cardiac complications such as myocarditis or pericarditis (23) or thromboembolic events (24)). Moreover, an accurate serodiagnosis is critical to elucidate the potential role of SARS-CoV-2 Omicron infection in the recent series of severe hepatitis cases in children with unknown etiology (25, 26).

Since the detection rates of the evaluated NC-specific antibody assays were not significantly affected in individuals with putative primary Omicron infection, we thus propose to use NC-specific antibody assays for the serodiagnosis of previous SARS-CoV-2 infections in such cases. Furthermore, assessing NC-specific antibodies becomes even more critical for differentiating between natural and vaccine-derived seroreactivity in vaccinated subjects. However, anti-NC assays also display limitations, including a decrease in sensitivity due to antibody waning over time and assay-specific differences in the overall sensitivity (27). Therefore, after adaption to the Omicron variant, S-specific antibody assays might still augment the serological identification of past SARS-CoV-2 infections in unvaccinated individuals whose NC-specific antibodies have already become undetectable. In addition, such an adaption might restrengthen the S-specific antibody assays′ correlation with Omicron-specific neutralizing antibodies.

A limitation of this study was that the infection with the Omicron variant was confirmed by a variant-specific PCR only in nine of the 37 convalescents. However, when RT-PCR testing revealed an infection in these individuals, the Omicron variant was the dominant circulating strain in Austria (17). Moreover, we did observe a significant loss in the sensitivity of Anti-S and Anti-RBD immunoassays in a subanalysis of those nine convalescents with PCR-confirmed Omicron infections.

Furthermore, we acknowledge that our cohort of convalescents after putative primary Omicron infection was relatively small (e.g., due to the current high vaccination coverage and seroprevalence due to previous infections). Nonetheless, since we demonstrate that antibody levels quantified by Anti-Spike and Anti-RBD immunoassays displayed a weaker correlation with variant-specific neutralizing antibody titers, our data might still serve as “proof of principle” that the antigenic changes of the Omicron variant affect commercial antibody assays.

Indeed, this study on the performances of 20 commercial SARS-CoV-2 antibody assays in primary infection with the SARS-CoV-2 Omicron variant demonstrates that the diagnostic ability of the currently available S- and RBD-specific immunoassays, particularly of sVNTs, may be significantly impaired, calling for continued adaptation of these immunoassays pending forthcoming course of the pandemic.

## MATERIALS AND METHODS

### Samples from convalescent individuals.

The study included serum samples from 80 nonhospitalized SARS-CoV-2 convalescents. SARS-CoV-2 infection was confirmed in all these individuals by positive RT-PCR from nasopharyngeal swabs. In 37 of 80 (female *n* = 21, male *n* = 16, median age: 41 years, range: 4 to 81), SARS-CoV-2 infection was confirmed by PCR between January and March 2022, when the Omicron variant circulated with over 98% predominance in Austria (17). None of the subjects had been vaccinated or had had a positive SARS-CoV-2 test before. Furthermore, in 10 of the 37 individuals, a previously acquired serum sample (in December 2021) tested negative for Anti-SARS-CoV-2 antibodies (using the SARS-CoV-2 ViraChip IgG assay, Viramed Biotech AG, Planegg, Germany). Seroconversion was thus documented in these 10 convalescents, confirming SARS-CoV-2 primary infection. In addition, infection with Omicron BA.1 or BA.2 variants was assessed by variant-specific RT-PCR in nasopharyngeal swabs obtained from 9 of 37 subjects, using the mutation assay VirSNiP SARS-CoV-2 Spike S371L S373P (TIB MOLBIOL, Berlin, Germany), as described previously (9). The presence of S371LS373P and S371FS373P98 indicated infection with Omicron BA.1 (*n* = 4) and BA.2 (*n* = 5), respectively.

Forty-three convalescents (female *n* = 21, male *n* = 22, median age: 33 years, range: 16 to 96) served as controls. In these individuals, SARS-CoV-2 infection was confirmed by RT-PCR from nasopharyngeal swabs taken between February 2020 and December 2020, a period when only an ancestral WT strain of the early pandemic circulated in Austria. None of the control individuals was vaccinated against SARS-CoV-2 since the vaccination was not yet available when the serum samples were obtained. Clinical information (including vaccination status, previous SARS-CoV-2 infections, documentation, date of RT-PCR positivity, and absence of hospitalization) was recorded before anonymizing samples.

All samples used for this study were initially obtained for routine serological testing at the Center for Virology. Residual sample material was then anonymized and integrated into the Center of Virology’s sample bank for research using a protocol approved by the local ethics committee (EK 1035/2016, EK 1513/2016). The ethics committee of the Medical University of Vienna approved the study protocol (EK 2156/2019). Since all individuals consented that SARS-CoV-2-specific antibody testing was performed at the Center for Virology, and only anonymized samples were retested for this study, the local ethics committee concluded that no written consent of the convalescents was required for this evaluation of commercial antibody tests (EK 2156/2019).

### Live-virus neutralization test.

The NT was conducted as described previously (28–30). In brief, the serum samples were incubated at 37°C with 50 to 100 TCID50 of either WT (GISAID accession number EPI_ISL_438123 [28]), Delta (GISAID accession number EPI_ISL_4172121 [30]), BA.1 (GISAID accession number EPI_ISL_9110894 [29]), or BA.2 (GISAID accession number EPI_ISL_11110193 [29]) virus strains for 1 h. The mixture was then added to a monolayer of VeroE6 cells (ECACC 85020206). After 3 to 5 days, the NT titers were determined as the reciprocal dilution factor at which serum antibodies prevented a virus cytopathic effect (CPE). Serial dilutions ranged from 1:10 to 1:1280. NT titers ≥10 were considered positive.

### Commercial antibody assays.

The panel of evaluated antibody tests comprised 20 different commercial antibody assays. Detailed information on these assays, including the test principle, the detected immunoglobulin classes, the respective target antigens, the measuring unit, and the cutoff values, are shown in Table S2. All assays were performed according to the manufacturer’s instructions, using the protocols, dilutions, and cutoff values the manufacturers provide.

### Statistical analyses.

Data analysis was performed with GraphPad Prism 9.3.1. For each test, we recorded the number of samples that tested above the manufacturer’s threshold as positive and calculated the detection rate as the percentage of positive samples of the total number of samples in the respective cohort. Detection rates of each test in the two cohorts were compared using the two-tailed Fisher’s exact test, and the *P*-values were adjusted by Bonferroni correction for multiple testing for this analysis (Anti-S- and Anti-RBD tests, *n* = 16; Anti-NC assays, *n* = 8; and immunoblots, *n* = 4). The alpha level was set to 0.05. The quantitative results of the antibody assays were plotted versus the Omicron NT-titers (using the higher titer of either the BA.1- or BA.2-specific NT) for the omicron cohort and against the WT NT titers for the WT cohort with a linear regression line. The correlation was assessed by calculating the Pearson correlation coefficient (r).
