# Supplementary material for: Reduced Sensitivity of Commercial Spike-Specific Antibody Assays after Primary Infection with the SARS-CoV-2 Omicron Variant
Source: Microbiol Spectr. 2022 Aug 25;10(5):e02129-22. doi: 10.1128/spectrum.02129-22 (PMC9603218; doi:10.1128/spectrum.02129-22)
Supplement: Supplemental file 1 — Fig. S1 to S9 and Tables S1 to S3. Download spectrum.02129-22-s0001.pdf, PDF file, 1.4 MB [file spectrum.02129-22-s0001.pdf]

Supplementary Information

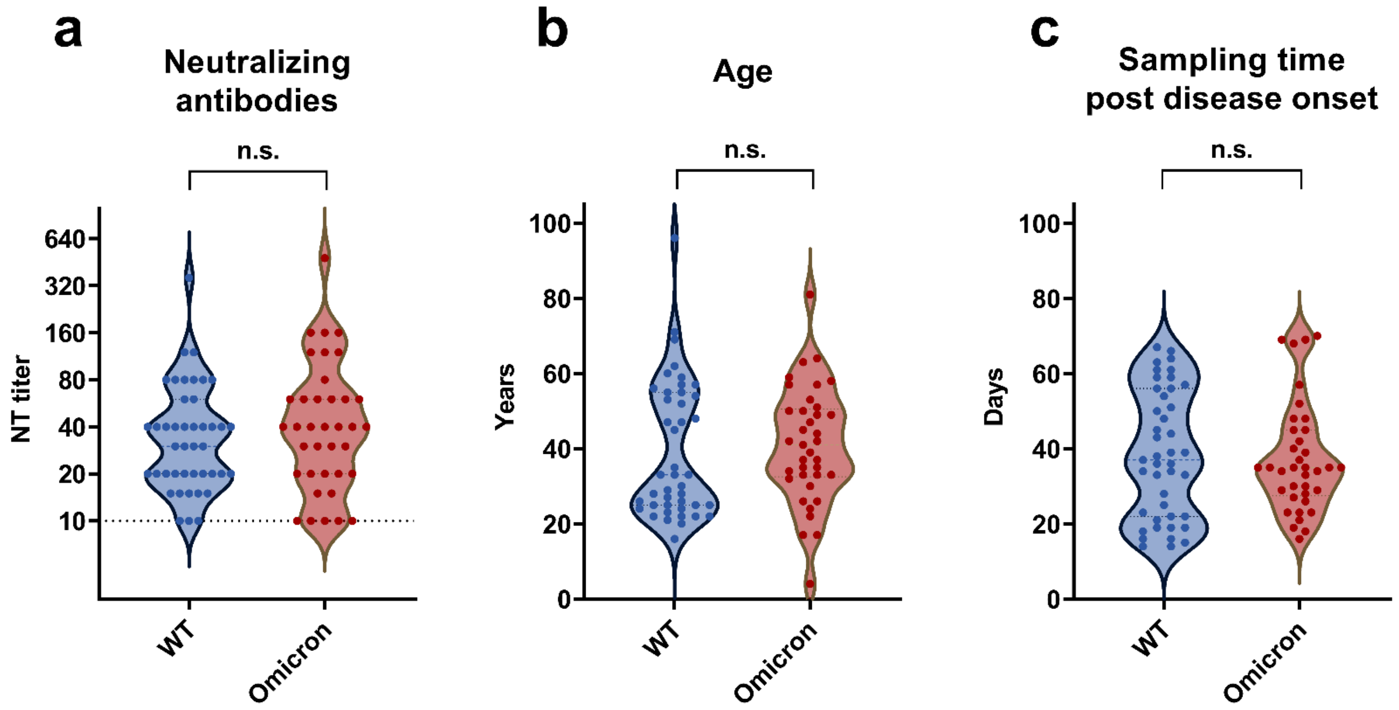

Supplementary Figure S1: Matching of the two cohorts according to NT titers, age, and sampling time points

Blue dots: Control cohort (n = 43, WT); red dots: omicron cohort (n = 37); a) Comparison of the WT-specific NT titer levels of the control cohort vs. the Omicron (BA.1/BA.2) NT titer levels. No significant difference in a two-tailed Mann-Whitney U test (p = 0.42). b) Comparison of the age of the two cohorts at the time of PCR positivity (not significant, p = 0.32; Mann Whitney U test as above); c) Comparison of the time between disease onset (defined as the first positive PCR test) and the blood sampling of the two cohorts (not significant, p = 0.86; Mann Whitney U test as above); n.s.: not significant (p > 0.05).

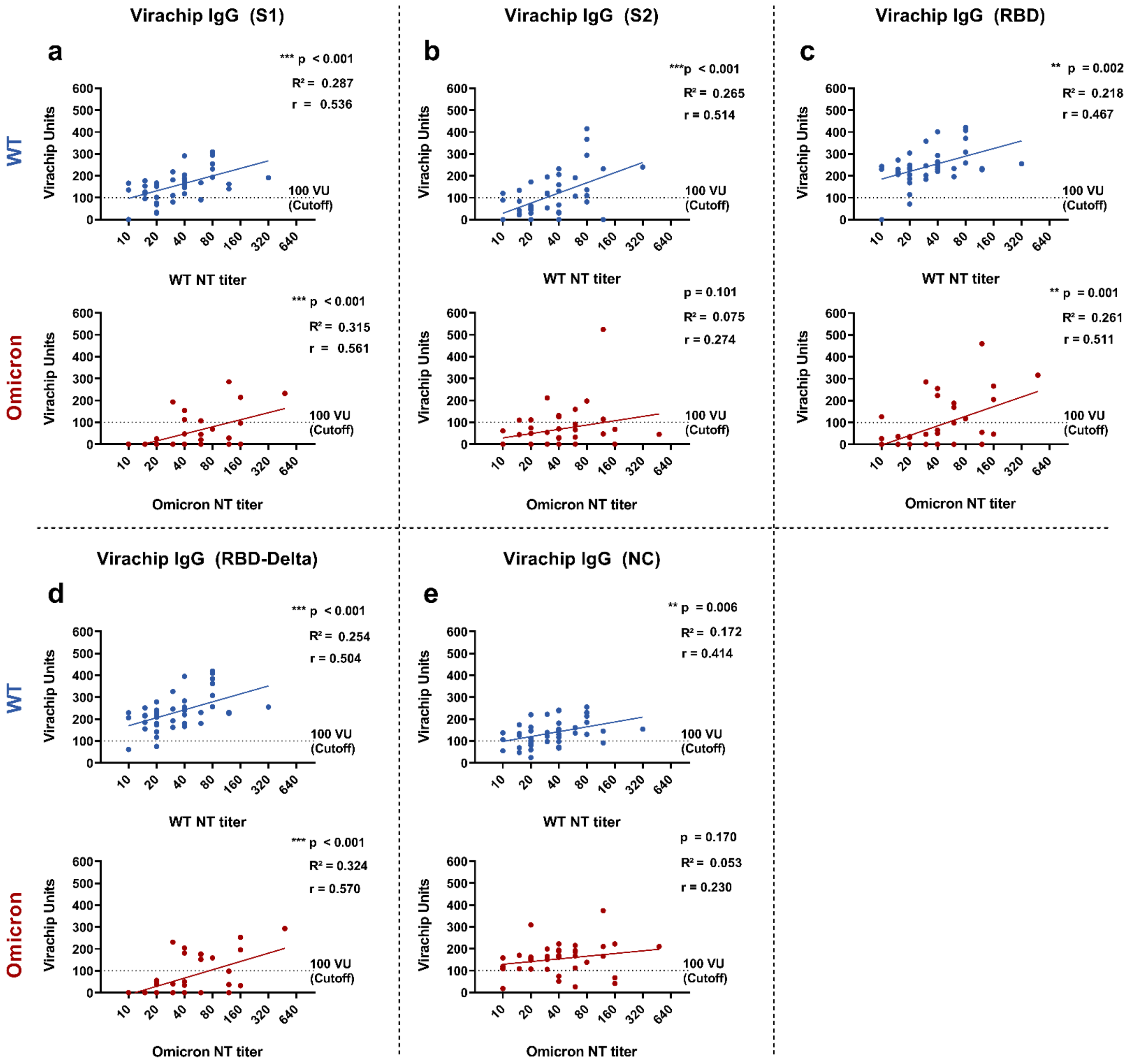

Supplementary Figure S2: Correlation of Virachip IgG microarray antibody levels with variant-specific NT titers in SARS-CoV-2 wildtype (WT) and putative primary Omicron infection

Graphical description of the correlation and the linear regression of the quantitative results by the Virachip IgG microarray in Virachip Units (VU) to the respective titers of variant-specific NTs (Omicron, WT; titer in log transformation). The Virachip IgG test assesses several target antigens simultaneously (S1, S2, RBD, RBD-Delta, and NC). a) S1 vs. WT NT (blue) and S1 vs. Omicron NT (red)). b) S2 vs. WT NT (blue) and S2 vs. Omicron NT (red) c) RBD vs. WT NT (blue) and RBD vs. Omicron NT (red) d) RBD-Delta vs. WT NT (blue) and RBD-Delta vs. Omicron NT (red) e) NC vs. WT NT (blue) and NC vs. Omicron NT (red). a-d: anti-S IgG assays; e: anti-NC IgG antibody assay. Dashed lines indicate the cutoff as recommended by the manufacturer. Blue dots: WT cohort (n = 43); red dots: Omicron cohort (n = 37). p-values, correlation coefficients r and R^2 were calculated using Pearson correlation (* $p < 0.05$; ** $p < 0.01$, *** $p < 0.001$).

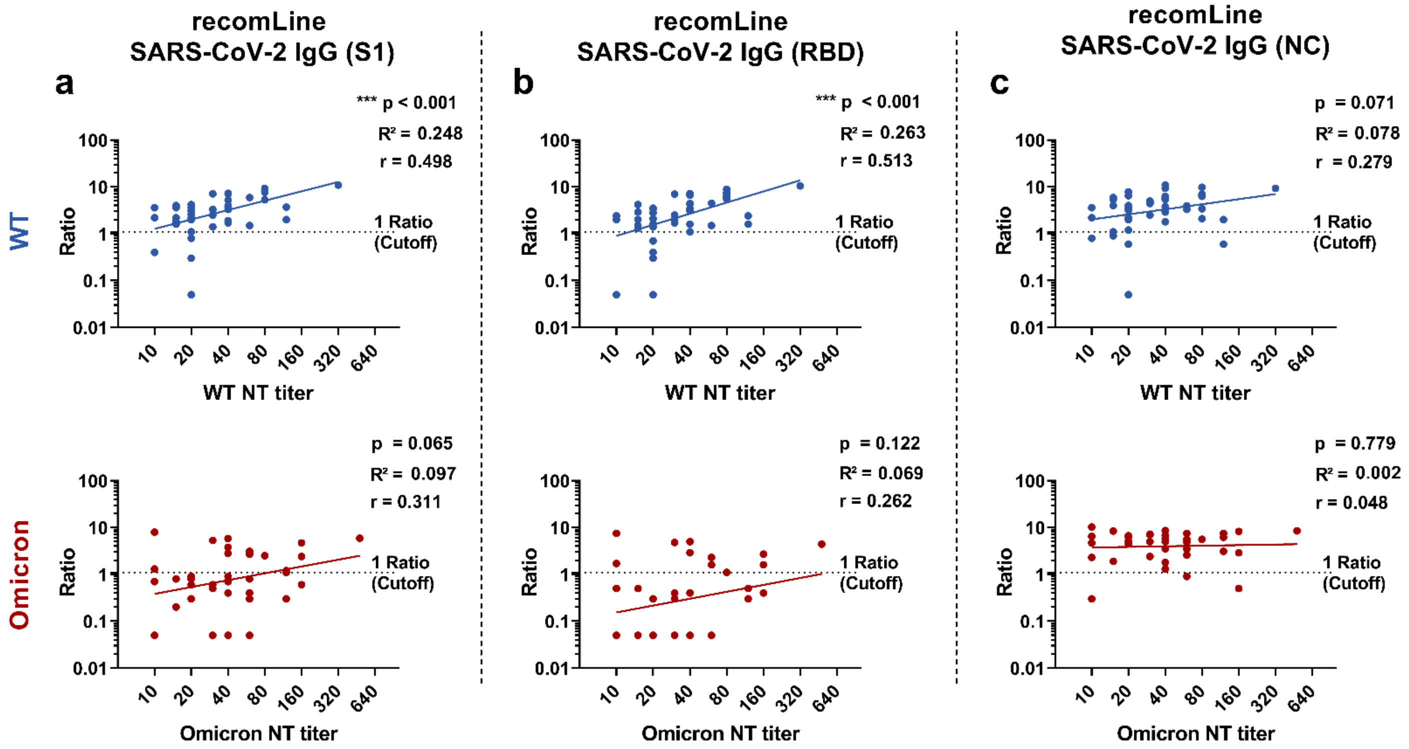

Supplementary Figure S3: Correlation of recomLine SARS-COV-2 IgG immunoblot antibody levels with variant-specific NT titers in SARS-CoV-2 wildtype (WT) and putative primary Omicron infection

Graphical description of the correlation and the linear regression of the quantitative results by the recomLine IgG immunoblot as (cutoff-) Ratio to the respective titers of variant-specific NTs (Omicron, WT; both in log transformation). The recomLine IgG test assesses several target antigens simultaneously (S1, RBD, and NC). a) S1 vs. WT NT (blue) and S1 vs. Omicron NT (red)). b) RBD vs. WT NT (blue) and RBD vs. Omicron NT (red) c) NC vs. WT NT (blue) and NC vs. Omicron NT (red) a and b: anti-S IgG assays; c: anti-NC IgG antibody assay. Dashed lines indicate the cutoff as recommended by the manufacturer. Blue dots: WT cohort (n = 43); red dots: Omicron cohort (n = 36). p-values, correlation coefficients r and R^2 were calculated using Pearson correlation (* $p < 0.05$; ** $p < 0.01$, *** $p < 0.001$).

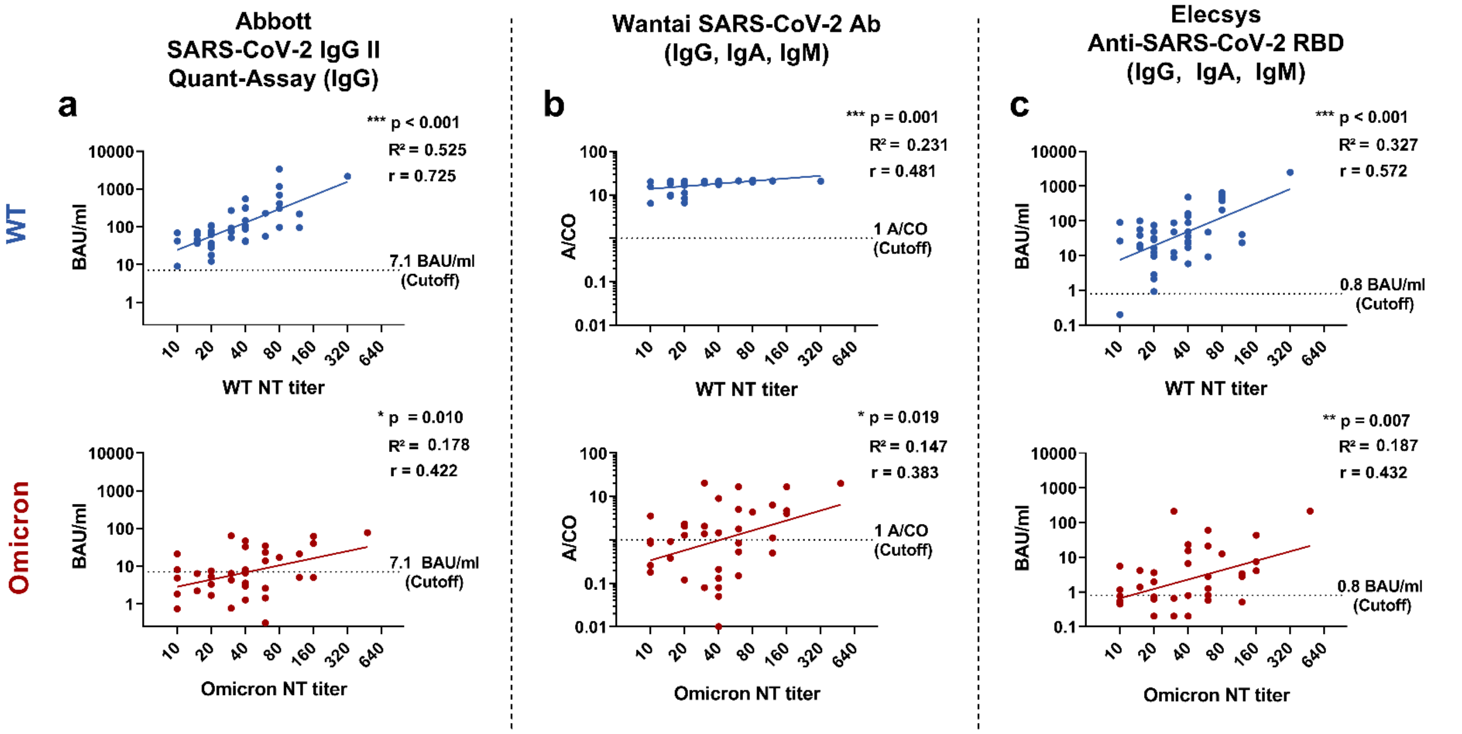

Supplementary Figure S4: Correlation of anti-Spike (S)- antibody levels with variant-specific NT titers in SARS-CoV-2 wildtype (WT) and putative primary Omicron infection

Graphical description of the correlation and the linear regression of the quantitative results of one Anti-IgG assay (Abbott) and two total antibody assays (Wantai, Elecsys) to the respective titers of variant-specific NTs (Omicron, WT; both in log transformation). a) SARS-CoV-2 IgG II Quant-Assay by Abbott (in BAU/ml) vs. WT NT (blue) and SARS-CoV-2 IgG II Quant-Assay vs. Omicron (red) b) Wantai SARS-CoV-2 Ab (in cutoff ratio (A/CO)) vs WT NT (blue) and Wantai SARS-CoV-2 Ab vs. Omicron NT (red) Dashed lines indicate the cutoff as recommended by the manufacturer. Blue dots: WT cohort (n = 43); red dots: Omicron cohort (n = 37), except for Abbott (panel a) where Omicron n = 36 (no sample material left for one sample). p-values, correlation coefficients r and R^2 were calculated using Pearson correlation (* $p < 0.05$; ** $p < 0.01$, *** $p < 0.001$).

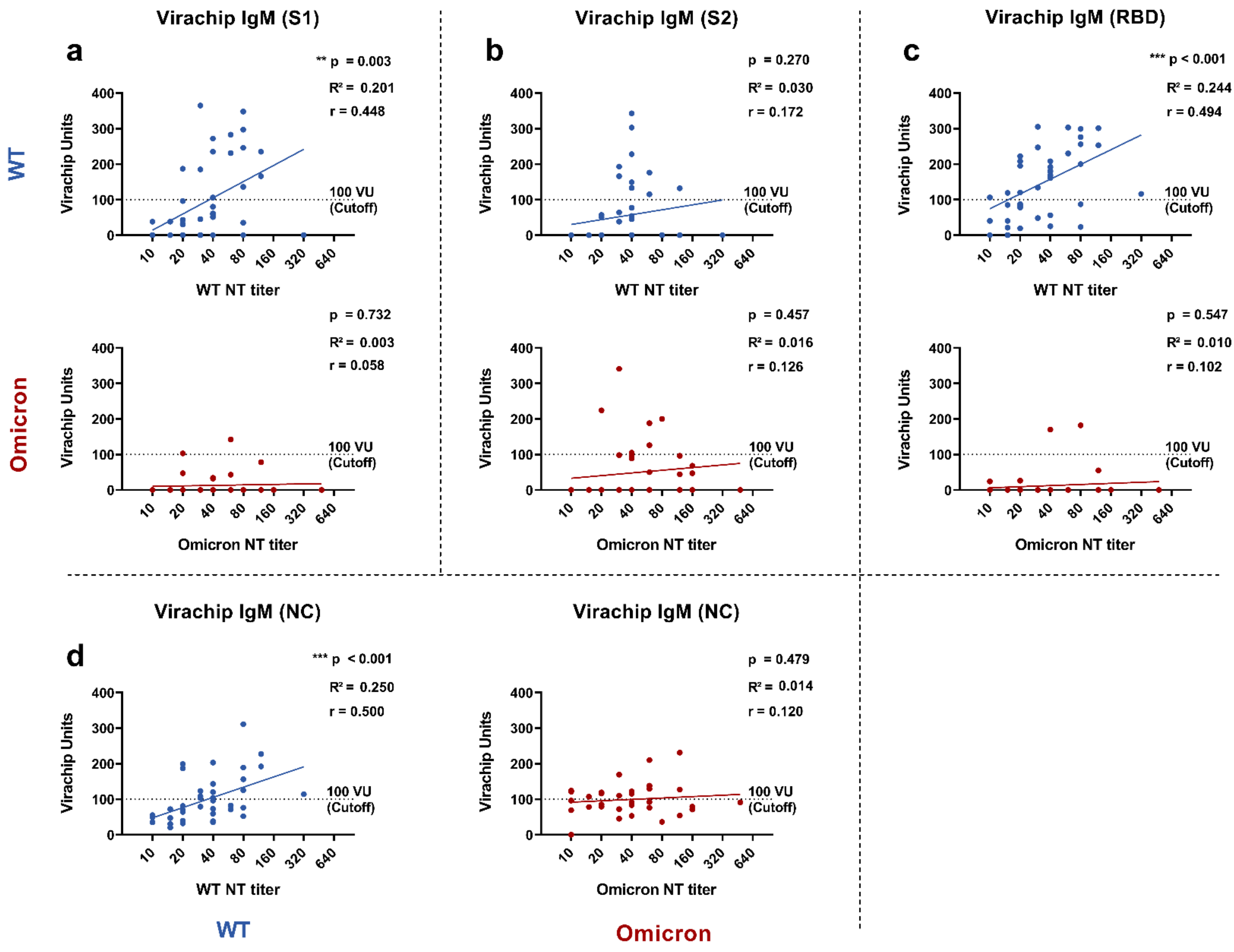

Supplementary Figure S5: Correlation of Virachip IgM microarray antibody levels with variant-specific NT titers in SARS-CoV-2 wildtype (WT) and putative primary Omicron infection

Graphical description of the correlation and the linear regression of the quantitative results by the Virachip IgM microarray in Virachip Units (VU) to the respective titers of variant-specific NTs (Omicron, WT; titer in log transformation). The Virachip IgM test assesses several target antigens simultaneously (S1, S2, RBD, and NC). a) S1 vs. WT NT (blue) and S1 vs. Omicron NT (red)). b) S2 vs. WT NT (blue) and S2 vs. Omicron NT (red) c) RBD vs. WT NT (blue) and RBD vs. Omicron NT (red) d) NC vs. WT NT (blue) and NC vs. Omicron NT (red). a-c: anti-S IgG assays; d: anti-NC IgG antibody assay. Dashed lines indicate the cutoff as recommended by the manufacturer. Blue dots: WT cohort (n = 43); red dots: Omicron cohort (n = 37). p-values, correlation coefficients r and R^2 were calculated using Pearson correlation (* $p < 0.05$; ** $p < 0.01$, *** $p < 0.001$).

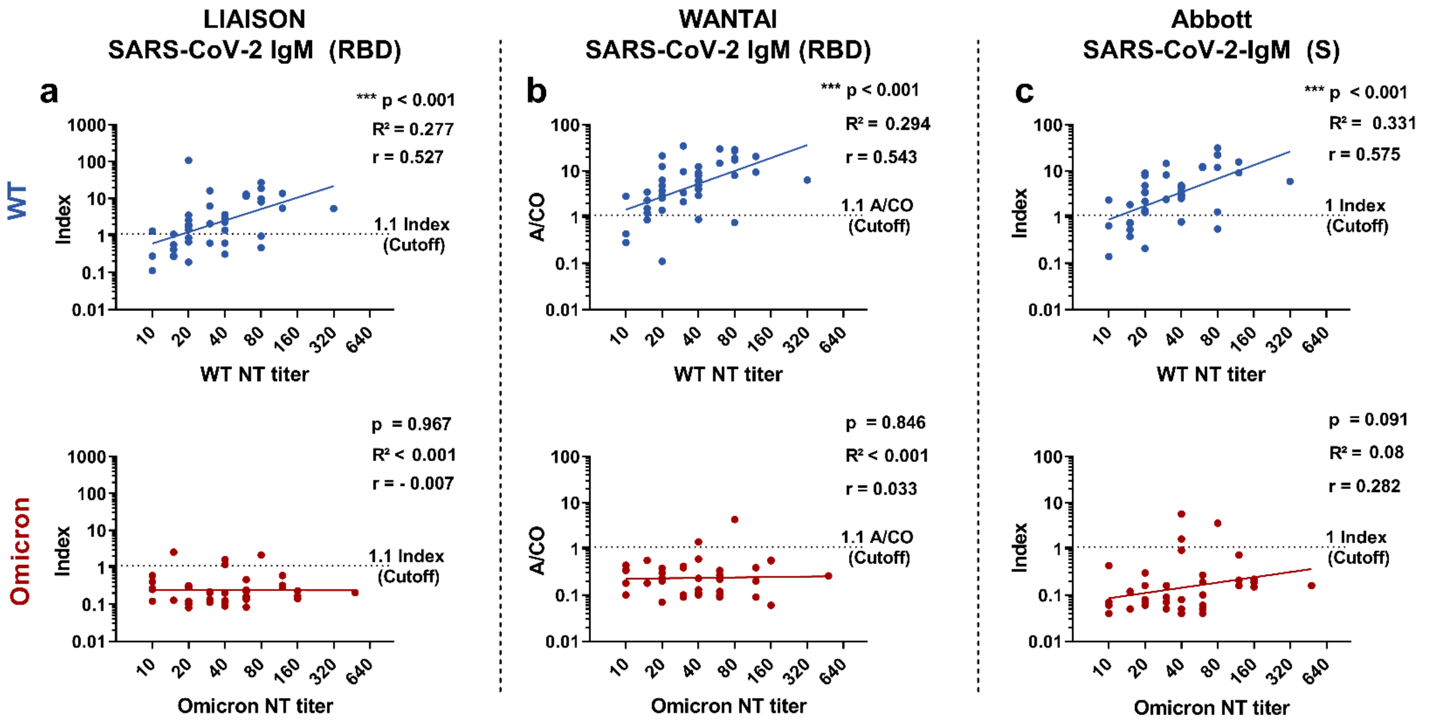

Supplementary Figure S6: Correlation of anti-Spike (S)- and anti-RBD IgM antibody levels with variant-specific NT titers in SARS-CoV-2 wildtype (WT) and putative primary Omicron infection

Graphical description of the correlation and the linear regression of the quantitative results of three Anti-IgM assays to the respective titers of variant-specific NTs (Omicron, WT; both in log transformation). a) Liaison SARS-CoV-2 IgM by DiaSorin (as (cutoff-) index) vs. WT NT (blue) and Liaison SARS-CoV-2 IgM vs. Omicron (red) b) Wantai SARS-CoV-2 IgM (in cutoff ratio (A/CO)) vs WT NT (blue) and Wantai SARS-CoV-2 vs. Omicron NT (red) c) SARS-CoV-2-IgM by Abbott (as (cutoff-) index) vs WT NT (blue) and SARS-CoV-2-IgM vs. Omicron NT (red). Dashed lines indicate the cutoff as recommended by the manufacturer. Blue dots: WT cohort (n = 43); red dots: Omicron cohort (n = 37). p-values, correlation coefficients r and R^2 were calculated using Pearson correlation (* $p < 0.05$; ** $p < 0.01$, *** $p < 0.001$).

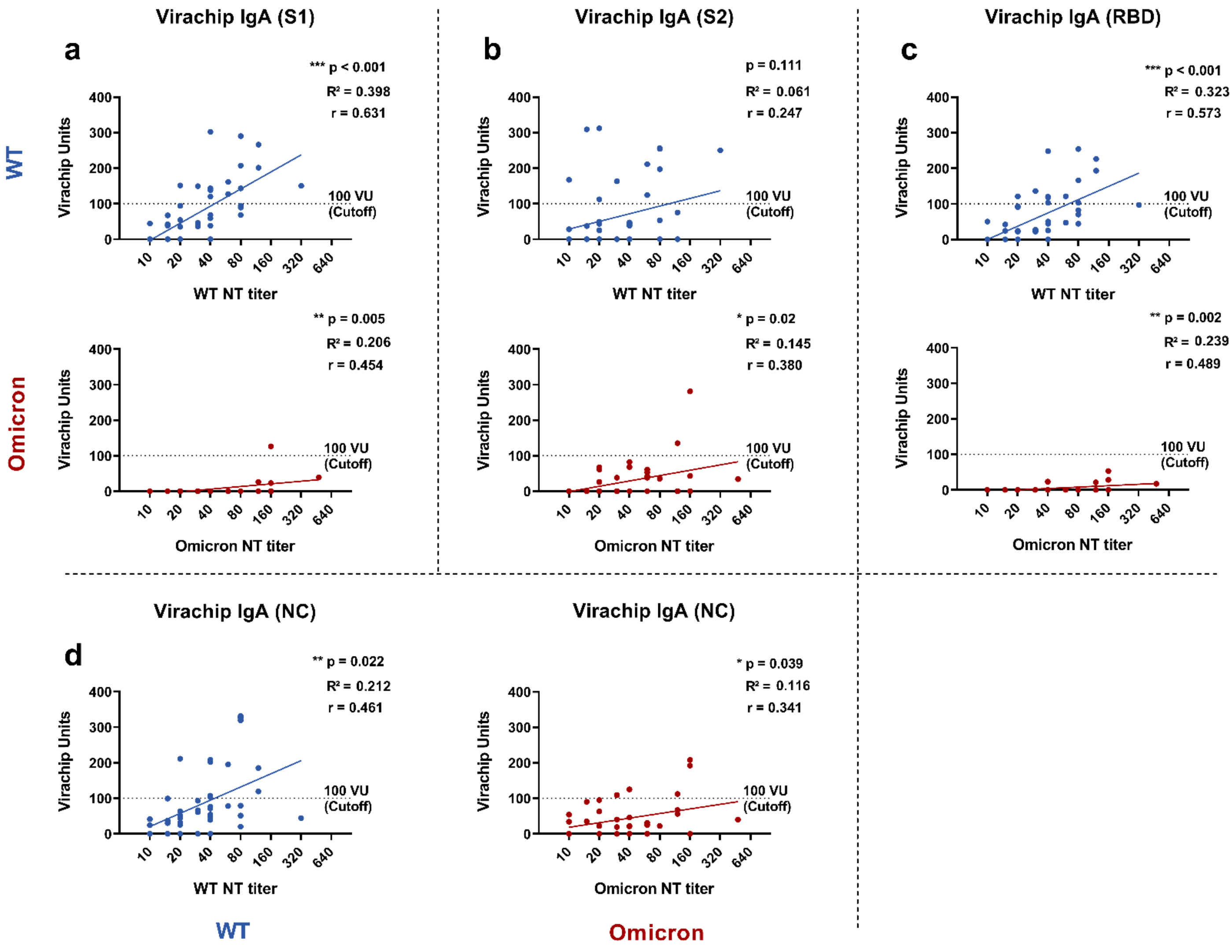

Supplementary Figure S7: Correlation of Virachip IgA microarray antibody levels with variant-specific NT titers in SARS-CoV-2 wildtype (WT) and Omicron primary infections

Graphical description of the correlation and the linear regression of the quantitative results by the Virachip IgA microarray in Virachip Units (VU) to the respective titers of variant-specific NTs (Omicron, WT; titer in log transformation). The Virachip IgA test assesses several target antigens simultaneously (S1, S2, RBD, and NC). a) S1 vs. WT NT (blue) and S1 vs. Omicron NT (red)). b) S2 vs. WT NT (blue) and S2 vs. Omicron NT (red) c) RBD vs. WT NT (blue) and RBD vs. Omicron NT (red) d) NC vs. WT NT (blue) and NC vs. Omicron NT (red). a-c: anti-S IgG assays; d: anti-NC IgG antibody assay. Dashed lines indicate the cutoff as recommended by the manufacturer. Blue dots: WT cohort (n = 43); red dots: Omicron cohort (n = 37). p-values, correlation coefficients r and R^2 were calculated using Pearson correlation (* $p < 0.05$; ** $p < 0.01$, *** $p < 0.001$).

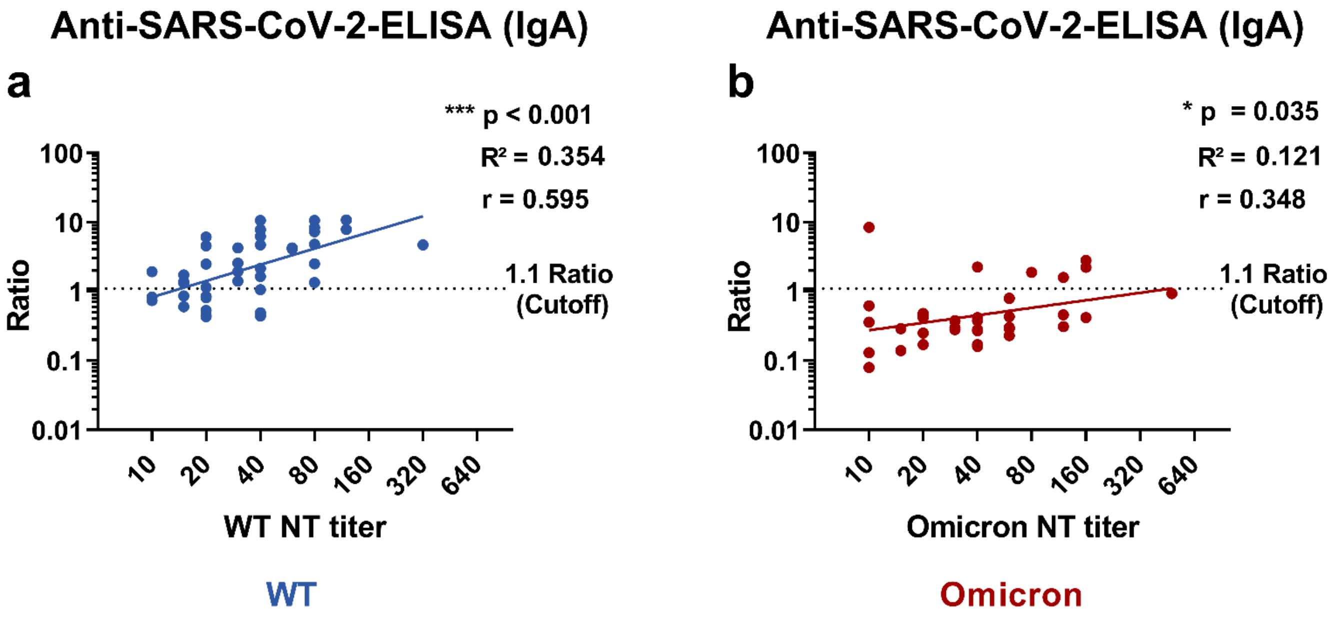

Supplementary Figure S8: Correlation of anti-Spike (S)-IgA antibody levels with variant-specific NT titers in SARS-CoV-2 wildtype (WT) and Omicron primary infections

Graphical description of the correlation and the linear regression of the quantitative results of the Anti-SARS-CoV-2-ELISA (IgA) by Euroimmun (in (cutoff-)ratio) to the respective titers of variant-specific NTs (Omicron, WT; both in log transformation). a) Anti-SARS-CoV-2-ELISA IgA vs. WT NT (blue) b) Anti-SARS-CoV-2-ELISA IgA vs. Omicron NT (red). Dashed lines indicate the cutoff as recommended by the manufacturer. Blue dots: WT cohort (n = 43); red dots: Omicron cohort (n = 37). p-values, correlation coefficients r and R^2 were calculated using Pearson correlation (* $p < 0.05$; ** $p < 0.01$, *** $p < 0.001$).

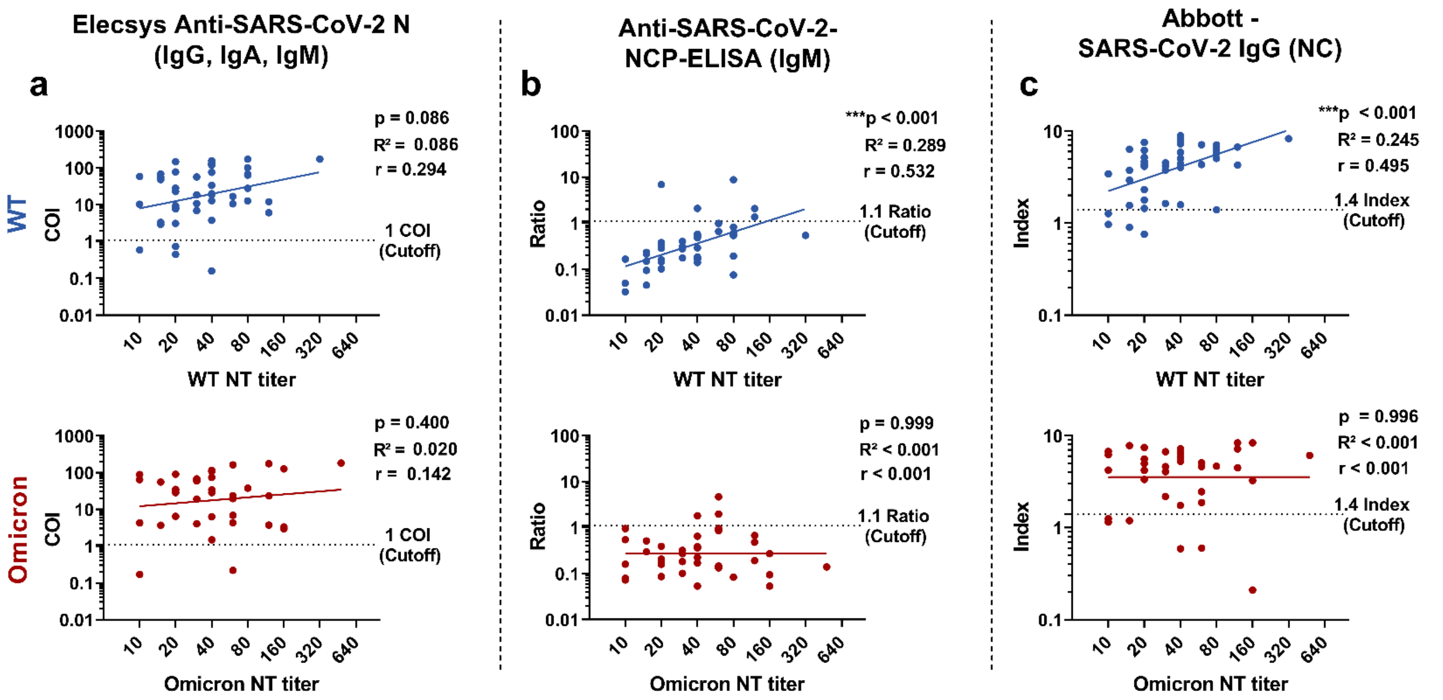

Supplementary Figure S9: Correlation of anti-Nucleocapsid (NC)-antibody levels with variant-specific NT titers in SARS-CoV-2 wildtype (WT) and Omicron primary infections

Graphical description of the correlation and the linear regression of the quantitative results of three Anti-Nucleocapsid assays (as cutoff ratio expressed as COI, ratio or index) to the respective titers of variant-specific NTs (Omicron, WT; both in log transformation). a) Elecsys Anti-SARS-CoV-2 N by Roche vs. WT NT (blue) and Elecsys Anti-SARS-CoV-2 N vs Omicron NT (red) b) Anti-SARS-CoV-2-NCP-ELISA (IgM) by Euroimmun vs. WT NT (blue) and Anti-SARS-CoV-2-NCP-ELISA (IgM) vs Omicron NT (red). C) SARS-CoV-2 IgG (NC) by Abbott vs. WT NT (blue) and SARS-CoV-2 IgG (NC) by Abbott vs. Omicron NT (red). Dashed lines indicate the cutoff as recommended by the manufacturer. Blue dots: WT cohort (n = 43); red dots: Omicron cohort (n = 37). p-values, correlation coefficients r and R^2 were calculated using Pearson correlation (* $p < 0.05$; ** $p < 0.01$, *** $p < 0.001$).

Supplemental Table S1: Evaluated antibody assays and cutoffs

**Abbott Laboratories, North Chicago, USA**	SARS-CoV-2 IgG assay	**CMIA**	**NC**	**Semi-Quant.**	**1.4 Index**
	SARS-CoV-2 IgM	CMIA	S	Semi-Quant.	1 Index
	SARS-CoV-2 IgG II Quant-Assay	CMIA	S	Quant.	7.1 BAU/ml
**Beijing Wantai, Beijing, China**	WANTAI SARS-CoV-2 Ab ELISA	ELISA	RBD	Semi-Quant.	1 A/CO
	WANTAI SARS-CoV-2 IgM ELISA	ELISA	RBD	Semi-Quant.	1.1 A/CO
**DiaSorin S.p.A, Saluggia, Italy**	LIAISON® SARS-CoV-2 IgM	CLIA	S1 + RBD	Quant.	1.1 Index
	LIAISON® SARS-CoV-2 TrimericS IgG assay	CLIA	S	Quant.	33.8 BAU/ml
**EUROIMMUN Medizinische Labordiagnostika AG, Lübeck, Germany**	Anti-SARS-CoV-2-QuantiVac-ELISA (IgG)	ELISA	S	Quant.	35.2 BAU/ml
	SARS-CoV-2-NeutraLISA	ELISA	RBD	Semi-Quant.	35%
	Anti-SARS-CoV-2-ELISA (IgA)	ELISA	S	Semi-Quant.	1.1 Ratio
	Anti-SARS-CoV-2-NCP-ELISA (IgG)	ELISA	NC	Semi-Quant.	1.1 Ratio
	Anti-SARS-CoV-2-NCP-ELISA (IgM)	ELISA	NC	Semi-Quant.	1.1 Ratio
**GenScript, New Jearsey, USA**	cPass ® SARS-CoV-2 Neutralization Antibody Detection Kit	sVNT	RBD-ACE2	Quant.	30 % IH
**MIKROGEN GMBH, Neuried, Germany**	recomLine SARS-CoV-2 IgG	Immunoblot	S1, RBD, NC	Qual.	1 signal ≥ 1Ratio
**Roche Diagnostics GmbH, Mannheim, Germany**	Elecsys Anti-SARS-CoV-2 N	ECLIA	NC	Semi-Quant.	1 COI
	Elecsys Anti-SARS-CoV-2 S	ECLIA	RBD	Quant.	0.8 BAU/ml
**TECOmedical AG, Sissach, Switzerland**	TECO® SARS-CoV-2 Neutralization Antibody Assay	sVNT	RBD-ACE2	Quant.	20 % IH
**Viramed Biotech AG, Planegg, Germany**	SARS-CoV-2 ViraChip ® IgG	Microarray	S1, RBD, RBDd, S2, NC	Quant.	1 signal ≥ 100 VU
	SARS-CoV-2 ViraChip ® IgM	Microarray	S1, RBD, S2, NC	Quant.	2 signals ≥100 VU
	SARS-CoV-2 ViraChip ® IgA	Microarray	S1, RBD, S2, NC	Quant.	2 signals ≥100 VU

sVNT surrogate virus neutralization test, ELISA enzyme-linked immunosorbent assay, CLIA chemiluminescence immunoassay, CMIA: chemiluminescence micro particle assay, ECLIA: electrochemiluminescence immunoassay, % IH percent inhibition, VU Virachip Units, Quant. quantative; Semi-quant semi-quantiative, S1 / S2 subunits S1 and S2 of the Spike protein, S Spike, RBD receptor-binding domain, RBDd receptor-binding domain of the Delta variant, NC nucleocapid protein

Supplementary Table S2: Re-calculation: only variant-PCR confirmed Omicron infection

	Target Antibodies	Test-Kit	Prin-ciple	Target Antigen(s)	WT n	WT %	Omicron n	Omicron %	Fisher p-value
		**Surrogate Virus Neutralization Test (sVNT)**							
Anti-S- / Anti-RBD antibody assay	IgG/A/M	**cPass** SARS-CoV-2 Neutralization Antibody Detection Kit **(GenScript)**	sVNT	RBD-ACE2 Inhibition	40/43	**93%**	2/9	**22%**	**< 0.0001**
	IgG/A/M	**TECO** SARS-CoV-2 Neutralization Antibody Assay **(TECOmedical)**	sVNT	RBD-ACE2 Inhibition	38/43	**88%**	2/9	**22%**	**0.0002**
	IgG/A/M	SARS-CoV-2-**NeutraLISA (Euroimmun)**	sVNT	RBD-ACE2 Inhibition	30/43	**70%**	1/9	**11%**	**0.0018**
		**Anti-S-Total Antibody Tests**							
	IgG/A/M	WANTAI **SARS-CoV-2 Ab Elisa (Wantai)**	ELISA	RBD	43/43	**100%**	5/9	**56%**	**0.0005**
	IgG/A/M	**Elecsys** Anti-SARS-CoV-2 **S (Roche)**	ECLIA	RBD	42/43	**98%**	6/9	**67%**	**0.0138**
		**Anti-S-IgG / Anti-RBD-IgG**							
	IgG	**SARS-CoV-2 IgG II Quant-Assay (Abbott)**	CMIA	S	43/43	**100%**	7/8	**88%**	0.1568
	IgG	SARS-CoV-2 **Virachip IgG (Viramed)**	MA	S1+S2+RBD +RBDd	42/43	**98%**	5/9	**56%**	**0.0228**
	IgG	**LIAISON** SARS-CoV-2 **TrimericS IgG** assay **(DiaSorin)**	CLIA	S	41/43	**95%**	6/9	**67%**	**0.0313**
	IgG	Anti-SARS-CoV-2-**QuantiVac**-ELISA **(Euroimmun)**	ELISA	S	39/43	**91%**	6/9	**67%**	0.0899
	IgG	**recomLine** SARS-CoV-2 IgG **(Mikrogen)**	IB	S1+RBD	39/43	**91%**	6/9	**67%**	**< 0.0001**
		**Anti-S-IgM / Anti-RBD-IgM**							
	IgM	**WANTAI** SARS-CoV-2 **IgM** Elisa **(Wantai)**	ELISA	RBD	37/43	**86%**	0/9	**0%**	**< 0.0001**
	IgM	**SARS-CoV-2 IgM (Abbott)**	CMIA	S	33/43	**77%**	0/9	**0%**	**< 0.0001**
	IgM	SARS-CoV-2 **Virachip IgM (Viramed)**	MA	S1+S2+RBD	27/43	**63%**	2/9	**22%**	0.061
	IgM	**LIAISON** SARS-CoV-2 **IgM (DiaSorin)**	CLIA	RBD	26/43	**60%**	1/9	**11%**	**< 0.0001**
		**Anti-S-IgA / Anti-RBD-IgA**							
	IgA	**Anti-SARS-CoV-2-ELISA (IgA) (Euroimmun)**	ELISA	S	31/43	**72%**	1/9	**11%**	**0.0011**
	IgA	SARS-CoV-2 **Virachip IgA (Viramed)**	MA	S1+S2+RBD	20/43	**47%**	0/9	**0%**	**0.0088**
		**Anti-NC-Antibody Assays (IgG, IgA, IgM)**							
Anti-NC-antibody assay	IgG/A/M	**Elecsys** Anti-SARS-CoV-2 **N (Roche)**	ECLIA	NC	39/43	**91%**	9/9	**100%**	> 0.9999
	IgG	**SARS-CoV-2 IgG (Abbott)**	CMIA	NC	38/43	**88%**	9/9	**100%**	0.5726
	IgG	**recomLine** SARS-CoV-2 IgG **(Mikrogen)**	IB	NC	38/43	**88%**	9/9	**100%**	0.5726
	IgG	**Anti-SARS-CoV-2-NCP**-ELISA **(IgG) (Euroimmun)**	ELISA	NC	35/43	**81%**	9/9	**100%**	0.323
	IgG	SARS-CoV-2 **Virachip IgG (Viramed)**	MA	NC	29/43	**67%**	9/9	**100%**	0.092
	IgM	SARS-CoV-2 **Virachip IgM (Viramed)**	MA	NC	16/43	**37%**	4/9	**44%**	0.7192
	IgM	**Anti-SARS-CoV-2-NCP**-ELISA **(IgM) (Euroimmun)**	ELISA	NC	5/43	**11%**	2/9	**22%**	0.5899
	IgA	SARS-CoV-2 **Virachip IgA (Viramed)**	MA	NC	11/43	**26%**	1/9	**11%**	0.6655

For this table, only Omicron samples were included in which a variant-specific RT-PCR was available (BA.1 n = 4; BA.2 n = 5) and compared to the WT cohort. N number of positive samples/ number of samples tested, % percent of positive samples, Fisher's exact test (two-tailed, alpha level = 0.05), sVNT SARS-CoV-2 Surrogate Virus Neutralization Tests, ELISA enzyme-linked immunosorbent assay; CLIA chemiluminescence immunoassay; CMIA chemiluminescence micro particle assay; ECLIA electrochemoluminescence immunoassay, IB immunoblot, S spike, S1/S2 subunit 1/2 of spike, RBD receptor-binding-domain; RBDd receptor binding domain of the Delta variant, NC nucleocapsid, MA: microarray. WT: n = 43; Omicron: n = 9; except for SARS-CoV-2 IgG II Quant-Assay (Abbott) where Omicron n = 8 (no sample material left in one sample). Significant p-values ($p < 0.05$): bold, underlined.

Supplementary Table S3: Re-analysis of the results comparing only samples with NT ≥ 20

	Target Antibodies	Test-Kit	Prin-ciple	Target Antigen(s)	WT NT ≥ 20		Omicron NT ≥ 20		Fisher
					**n**	**%**	**n**	**%**	**p-value**
**Anti-S- / Anti-RBD antibody assay**	**Surrogate Virus Neutralization Test (sVNT)**								
	IgG/A/M	**cPass** SARS-CoV-2 Neutralization Antibody Detection Kit **(GenScript)**	sVNT	RBD-ACE2 Inhibition	32/35	**91%**	2/30	**5%**	**< 0.0001**
		**TECO** SARS-CoV-2 Neutralization Antibody Assay **(TECOmedical)**	sVNT		30/35	**86%**	2/30	**7%**	**< 0.0001**
		SARS-CoV-2-**NeutraLISA (Euroimmun)**	sVNT		24/35	**69%**	1/30	**3%**	**< 0.0001**
	**Anti-S-Total Antibody Tests**								
	IgG/A/M	WANTAI **SARS-CoV-2 Ab Elisa (Wantai)**	ELISA	RBD	35/35	**100%**	18/30	**60%**	**< 0.0001**
		**Elecsys** Anti-SARS-CoV-2 **S (Roche)**	ECLIA		34/35	**97%**	17/30	**57%**	**0.0001**
	**Anti-S-IgG / Anti-RBD-IgG**								
	IgG	**SARS-CoV-2 IgG II Quant-Assay (Abbott)**	CMIA	S	35/35	**100%**	14/29	**48%**	**< 0.0001**
		SARS-CoV-2 **Virachip IgG (Viramed)**	MA	S1+S2+RBD +RBDd	35/35	**100%**	16/30	**53%**	**< 0.0001**
		**LIAISON** SARS-CoV-2 **TrimericS IgG** assay **(DiaSorin)**	CLIA	S	22/35	**63%**	3/30	**10%**	**< 0.0001**
		Anti-SARS-CoV-2-**QuantiVac**-ELISA **(Euroimmun)**	ELISA		32/35	**91%**	12/30	**40%**	**< 0.0001**
		**recomLine** SARS-CoV-2 IgG **(Mikrogen)**	IB	S1+RBD	28/35	**80%**	12/29	**40%**	**0.002**
	**Anti-S-IgM / Anti-RBD-IgM**								
	IgM	**WANTAI** SARS-CoV-2 **IgM** Elisa **(Wantai)**	ELISA	RBD	29/35	**83%**	2/30	**7%**	**< 0.0001**
		**SARS-CoV-2 IgM (Abbott)**	CMIA	S	29/35	**83%**	3/30	**10%**	**< 0.0001**
		SARS-CoV-2 **Virachip IgM (Viramed)**	MA	S1+S2+RBD	23/35	**66%**	8/30	**27%**	**0.0026**
		**LIAISON** SARS-CoV-2 **IgM (DiaSorin)**	CLIA	RBD	21/35	**60%**	3/30	**10%**	**< 0.0001**
	**Anti-S-IgA / Anti-RBD-IgA**								
	IgA	**Anti-SARS-CoV-2-ELISA (IgA) (Euroimmun)**	ELISA	S	18/35	**51%**	2/30	**7%**	**< 0.0001**
		SARS-CoV-2 **Virachip IgA (Viramed)**	MA	S1+S2+RBD	26/35	**74%**	5/30	**17%**	**0.0001**
**Anti-NC-antibody assay**	**Anti-NC-Antibody Assays (IgG, IgA, IgM)**								
	IgG/A/M	**Elecsys** Anti-SARS-CoV-2 **N (Roche)**	ECLIA	NC	32/35	**91%**	29/30	**97%**	>0.9999
	IgG	**SARS-CoV-2 IgG (Abbott)**	CMIA		30/35	**86%**	27/30	**90%**	0.7157
		**recomLine** SARS-CoV-2 IgG **(Mikrogen)**	IB		28/35	**80%**	27/29	**93%**	0.166
		**Anti-SARS-CoV-2-NCP**-ELISA **(IgG) (Euroimmun)**	ELISA		29/35	**83%**	27/30	**90%**	0.4883
		SARS-CoV-2 **Virachip IgG (Viramed)**	MA		24/35	**69%**	25/30	**83%**	0.249
	IgM	SARS-CoV-2 **Virachip IgM (Viramed)**	MA		11/35	**31%**	13/30	**43%**	0.44
		**Anti-SARS-CoV-2-NCP**-ELISA **(IgM) (Euroimmun)**	ELISA		2/35	**6%**	2/30	**7%**	>0.9999
	IgA	SARS-CoV-2 **Virachip IgA (Viramed)**	MA		9/35	**26%**	5/30	**17%**	0.5464

Comparison of detection rates in the Omicron vs. WT cohorts, only including samples with high NT titer (≥ 20). N number of positive samples/ number of samples tested, % percent of positive samples, Fisher's exact test (two-tailed, alpha level = 0.05), sVNT SARS-CoV-2 Surrogate Virus Neutralization Tests, ELISA enzyme-linked immunosorbent assay; CLIA chemiluminescence immunoassay; CMIA chemiluminescence micro particle assay; ECLIA electrochemoluminescence immunoassay, IB immunoblot, S spike, S1/S2 subunit 1/2 of spike, RBD receptor-binding-domain; RBDd receptor binding domain of the Delta variant, NC nucleocapsid, MA: microarray. WT: n = 35; Omicron: n = 30; except for recomLine SARS-CoV-2 IgG (Mikrogen) and SARS-CoV-2 IgG II Quant-Assay (Abbott) where Omicron n = 29 (sample technically invalid and no material left respectively). Significant p-values ($p < 0.05$): bold, underlined.
